# Cardiovascular measures display robust phenotypic stability across long-duration intervals involving repeated sleep deprivation and recovery

**DOI:** 10.3389/fnins.2023.1201637

**Published:** 2023-07-20

**Authors:** Lauren N. Pasetes, Kathleen M. Rosendahl-Garcia, Namni Goel

**Affiliations:** ^1^Biological Rhythms Research Laboratory, Department of Psychiatry and Behavioral Sciences, Rush University Medical Center, Chicago, IL, United States; ^2^Siemens Healthineers, Mountain View, CA, United States

**Keywords:** hemodynamics, sleep deprivation, cardiovascular, individual differences, echocardiography, intraclass correlation coefficient, recovery, phenotype

## Abstract

**Introduction:**

We determined whether cardiovascular (CV) measures show trait-like responses after repeated total sleep deprivation (TSD), baseline (BL) and recovery (REC) exposures in two long-duration studies (total *N* = 11 adults).

**Methods:**

A 5-day experiment was conducted twice at months 2 and 4 in a 4-month study (*N* = 6 healthy adults; 3 females; mean age ± SD, 34.3 ± 5.7 years; mean BMI ± SD, 22.5 ± 3.2 kg/m^2^), and three times at months 2, 4, and 8 in an 8-month study (*N* = 5 healthy adults; 2 females; mean age ± SD, 33.6 ± 5.17 years; mean BMI ± SD, 27.1 ± 4.9 kg/m^2^). Participants were not shift workers or exposed to TSD in their professions. During each experiment, various seated and standing CV measures were collected via echocardiography [stroke volume (SV), heart rate (HR), cardiac index (CI), left ventricular ejection time (LVET), and systemic vascular resistance index (SVRI)] or blood pressure monitor [systolic blood pressure (SBP)] after (1) two BL 8h time in bed (TIB) nights; (2) an acute TSD night; and (3) two REC 8–10 h TIB nights. Intraclass correlation coefficients (ICCs) assessed CV measure stability during BL, TSD, and REC and for the BL and REC average (BL + REC) across months 2, 4, and 8; Spearman’s rho assessed the relative rank of individuals’ CV responses across measures.

**Results:**

Seated BL (0.693–0.944), TSD (0.643–0.962) and REC (0.735–0.960) CV ICCs showed substantial to almost perfect stability and seated BL + REC CV ICCs (0.552–0.965) showed moderate to almost perfect stability across months 2, 4, and 8. Individuals also exhibited significant, consistent responses within seated CV measures during BL, TSD, and REC. Standing CV measures showed similar ICCs for BL, TSD, and REC and similar response consistency.

**Discussion:**

This is the first demonstration of remarkably robust phenotypic stability of a number of CV measures in healthy adults during repeated TSD, BL and REC exposures across 2, 4, and 8 months, with significant consistency of responses within CV measures. The cardiovascular measures examined in our studies, including SV, HR, CI, LVET, SVRI, and SBP, are useful biomarkers that effectively track physiology consistently across long durations and repeated sleep deprivation and recovery.

## 1. Introduction

Chronic sleep deprivation is a prominent public health issue associated with numerous adverse health risks including cardiovascular (CV) disease, diabetes, cancer, obesity, morbidity, mortality, and Alzheimer’s disease (Ferrie et al., 2007; Gallicchio and Kalesan, 2009; Mullington et al., 2009; Niu et al., 2022). In response to total sleep deprivation (TSD) or sleep restriction (SR), CV measures such as cardiac index/cardiac output (CI/CO) (Sunbul et al., 2014; Papacocea et al., 2019; Yamazaki et al., 2022b), stroke volume (SV) (Lü et al., 2018; Papacocea et al., 2019; Yamazaki et al., 2022b), systemic vascular resistance index (SVRI) (Kato et al., 2000; Lü et al., 2018; Yamazaki et al., 2022b), systolic blood pressure (SBP) (Kato et al., 2000; Muenter et al., 2000; Meier-Ewert et al., 2004; Zhong et al., 2005; Mullington et al., 2009; Sauvet et al., 2010; Lü et al., 2018; Słomko et al., 2018; Kuetting et al., 2019; Papacocea et al., 2019; Bourdillon et al., 2021; Bozer et al., 2021; Cernych et al., 2021; Yamazaki et al., 2022b), heart rate (HR) (Kato et al., 2000; Meier-Ewert et al., 2004; Zhong et al., 2005; Mikulski et al., 2006; Sauvet et al., 2010; Sunbul et al., 2014; Keramidas et al., 2018; Lü et al., 2018; Kuetting et al., 2019; Bourdillon et al., 2021; Yamazaki et al., 2022b; Chamorro et al., 2023), and left ventricular ejection time (LVET) (Yamazaki et al., 2022b) have shown inconsistent findings, with some studies reporting alterations, while others show no changes. Although a number of studies have examined the effects of sleep loss on CV measures, only a few studies have assessed the stability of these measures with or without sleep loss.

Research has shown short-term and long-term stability in CV measures across repeated collections without sleep loss including HR (Woodward et al., 1995; Rowland et al., 1998; Goedhart et al., 2006), SV (Rowland et al., 1998; Goedhart et al., 2006; Legendre et al., 2018), CI/CO (Mier et al., 1997; Rowland et al., 1998; Goedhart et al., 2006), LVET (Goedhart et al., 2006), and SBP (Woodward et al., 1995; Mier et al., 1997; Hinderliter et al., 2013). Short-term stability of HR, SV, CO, LVET, and SBP, with intraclass correlation coefficients (ICCs) ranging from 0.030 to 0.930 across 2 h to 10 days, were demonstrated in healthy males and females (Woodward et al., 1995; Mier et al., 1997; Goedhart et al., 2006); SBP ICCs ranging from 0.420 to 0.630 were found across 3 weeks in males and females (Hinderliter et al., 2013). Long-term stability of SV, HR, LVET, and CI/CO with ICCs ranging from 0.290 to 0.920 across 2–40 months occurred in healthy males and females (Rowland et al., 1998; Goedhart et al., 2006; Legendre et al., 2018).

Although short-term and long-term stability of CV measures without sleep loss have been observed, only one study has examined CV stability during sleep loss: HR showed long-term stability (ICC: 0.790) after exposure to 26 h TSD twice across 2.5–15 months (Chua et al., 2014). To the best of our knowledge, both short-term and long-term stability in other cardiovascular measures including SBP, CO, SV, SVRI, and LVET have yet to be examined after exposure to TSD and no studies have examined the stability of CV measures during baseline and recovery phases surrounding sleep loss.

In this study, for the first time, we sought to address several important gaps in prior research with the following aims: (1) To determine the stability of various seated and standing CV measures across long-duration time points during acute TSD (2, 4 and 8 months); (2) To compare the long-term stability of various seated and standing CV measures across BL (2, 4, and 8 months) and across REC (2, 4, and 8 months); (3) To determine the stability of seated and standing CV measures across the average of BL and REC (BL + REC; 2, 4, and 8 months); and (4) To examine the relative rank of individuals across seated and standing CV measures at BL, TSD, and REC (2 and 4 months). We hypothesized CV measures would show high ICCs during BL, TSD, and REC phases across 2, 4, and 8 months. We also predicted that CV measures would show robust stability across BL + REC (2, 4, and 8 months). Finally, we hypothesized that in terms of relative rank, most CV measures would significantly relate to one another at BL, TSD, and REC.

## 2. Materials and methods

### 2.1. Participants

Nazemnyy Eksperimental’nyy Kompleks (NEK), located in the Institute of Biomedical Problems (IBMP) of the Russian Academy of Sciences, Moscow, Russia, is an isolation facility designed to conduct research studies examining the effects of spaceflight on behavioral health and performance (Cromwell et al., 2021; Anikushina et al., 2022; Le Roy et al., 2023). We conducted a 4-month study (*N* = 6 healthy adults; 3 females; mean age ± SD, 34.3 ± 5.7 years; mean BMI ± SD, 22.5 ± 3.2 kg/m^2^) from March 2019-July 2019, and a similar 8-month study (*N* = 5 healthy adults; 2 females; mean age ± SD, 33.6 ± 5.17 years; mean BMI ± SD, 27.1 ± 4.9 kg/m^2^) from November 2021-July 2022 in the IMBP NEK facility (Anikushina et al., 2022; Bell et al., 2023). Participants were not shift workers or exposed to TSD in their professions. They had strong technical and/or scientific backgrounds and human-support skills relevant for space exploration. Across the two studies, the nationalities of the participants were Russian (*N* = 6), American (*N* = 4), and Emirati (*N* = 1). Participants were screened thoroughly by the National Aeronautics and Space Administration (NASA). Inclusion/exclusion criteria required that the participants come from various cultures and nationalities, have no prior experience with space flight, and include both males and females; all participants passed a drug screen and a physical exam ensuring they were in excellent health with no history of neurological, CV, integumentary, musculoskeletal, or gastrointestinal problems, and passed a psychological assessment (Yamazaki et al., 2021, 2022b; Abeln et al., 2022; Saveko et al., 2022). The studies were approved by the Institutional Review Board of the NASA (4-month study and 8-month study) with primary oversight, and also by the Institutional Review Boards of the University of Pennsylvania (4-month study) and Rush University Medical Center (8-month study). All protocol methods were carried out according to approved regulations and guidelines. Participants provided written informed consent prior to inclusion in the study in accordance with the Declaration of Helsinki. Participants received compensation for their participation.

### 2.2. Procedures

During these NEK studies, a 5-day experimental protocol was conducted twice (at months 2 and 4) for the 4-month study, and a similar experimental protocol was conducted three times (at months 2, 4, and 8) for the 8-month study. The first two experiments (at months 2 and 4) occurred on the same days in the 4-month and 8-month studies. Participants received two nights of baseline sleep with 8-h time in bed (TIB) sleep opportunity (B1, B2; 2300 h–0700 h). Baseline CV measure collection occurred between approximately 0700 h–1200 h after the B2 night. Following B2 daytime, participants experienced continued wakefulness for approximately 39 h of TSD. During the TSD nights, participants were ambulatory. They engaged in a number of tasks to maintain wakefulness throughout the night during the TSD protocol, including completing various types of cognitive and operational performance tasks, team communications and decision-making tasks, and questionnaires and surveys. Participants also conducted various routine maintenance and resupply tasks in the facility. The last meal ended latest by 2200 h, to ensure a 9-h fasting period prior to CV collections the next morning (see below). During the TSD nights, participants were also monitored continuously by wrist actigraphy and by outside observers to ensure adherence. During TSD, CV measure collection occurred between approximately 0700 h–1200 h. Recovery sleep opportunities were 10 h TIB (R1; 2100 h–0700 h) and 8 h TIB (R2; 2300 h–0700 h). CV measure collection then occurred between approximately 0700 h–1200 h during R2, after the second recovery night of 8 h TIB. All assessments were conducted at the same time of day (in the morning before eating). All participants fasted for approximately 9 h or longer prior to all collections to maintain consistency across the study and among participants. Wrist actigraphy (Philips Respironics Healthcare, Bend, OR, USA) was used to measure and verify total sleep time during the 5-day experiments (Table 1). Actigraphic sleep data were analyzed as in our prior studies (e.g., Dennis et al., 2017; Moreno-Villanueva et al., 2018; Yamazaki and Goel, 2020; Brieva et al., 2021; Yamazaki et al., 2021, 2022a,b; Casale et al., 2022).

**TABLE 1:** Actigraphic total sleep time data during the 5-day experiments at month 2, at month 4, and at month 8 (mean ± SD).

		Month 2	Month 4	Month 8
*N*		11	11	5
Baseline 1	TST (min)	406.77 ± 33.98	421.68 ± 46.31	394.25 ± 37.87^b^
Baseline 2	TST (min)	398.61 ± 74.51	425.80 ± 44.51	424.00 ± 20.14
Total Sleep Deprivation	TST (min)	–	–	–
Recovery 1	TST (min)	534.36 ± 48.43*	568.18 ± 45.23^a^	535.80 ± 47.82
Recovery 2	TST (min)	354.39 ± 79.42	397.36 ± 34.90	355.20 ± 21.37

TST, total sleep time; ^a^*N* = 10; ^b^*N* = 4. *Month 2 TST was significantly shorter than month 4 TST (*t*(9) = –2.741, *P* = 0.023).

### 2.3. Cardiovascular measure collections

During these repeated experiments, CV measures were collected via echocardiography or blood pressure monitor (for SBP) at three assessment time points: (1) after two baseline 8h time in bed (TIB) nights (BL); (2) after a night of acute TSD; and (3) after two recovery nights of 8–10 h TIB (REC). SBP, SV, HR, CI, LVET, and SVRI were collected under highly controlled conditions. For the NEK 4-month study, echocardiography data were collected in both seated and standing positions, while SBP was only collected in the seated position. For the NEK 8-month study, echocardiography and SBP measures were collected in both seated and standing positions. As noted above, all collections were completed between 0700 and 1200 h.

#### 2.3.1. Echocardiogram procedures

Due to strict isolation conditions, one participant collected all cardiac ultrasound images on the other four or five participants during each study, and a second participant collected all cardiac ultrasound images on the primary collector during each study. All ultrasound operators were trained to collect ultrasound images and Doppler prior to the study and repeated identical collection procedures across each time point (Yamazaki et al., 2022b).

Stroke volume was collected via ultrasound imaging [GE Vivid q ultrasound system (General Electric Medical Systems, Milwaukee, WI, USA)] in both seated and standing postures at all time points (Ihlen et al., 1984; Haites et al., 1985; McLennan et al., 1986; Arbeille and Herault, 1998; Yamazaki et al., 2022b). Two-dimensional images of the left ventricular outflow tract (LVOT) were collected from each participant using a 5S-RS transducer (Yamazaki et al., 2022b). The LVOT was imaged from the parasternal long-axis view while the participants were semi-supine in a left lateral decubitus posture (Yamazaki et al., 2022b). Three to four, 2-s cine-loops of dynamic motion of the LVOT were digitally saved. SV was collected utilizing a continuous wave (CW) pencil (Pedof) probe for Doppler interrogation (Yamazaki et al., 2022b). CW Doppler signals were taken from the ascending aorta at the suprasternal notch in a seated and standing posture (Yamazaki et al., 2022b). Three 5-s cine-loop sweeps of CW Doppler data were collected and digitally stored as proprietary raw data (Yamazaki et al., 2022b).

Analysis of the digital data was conducted using Echo PAC PC (BT12) software (General Electric Medical Systems, Milwaukee, WI, USA). LVOT diameters were measured just proximal to the aortic valve leaflet insertion from three consecutive cine-loops at the maximum opening of the aortic valve. Five consecutive CW Doppler waveform profiles were traced to calculate the velocity time integral (VTI). The interval between each maximum peak on the Doppler spectral from the ascending aorta was used to calculate HR. The duration of each beat was measured to determine LVET for each SV. The VTI and LVET were then transferred from the Echo PAC software to Excel to calculate SV, HR, and CI using the following formulas:


SV=(LVOT⁢cross⁢sectional⁢area)×VTI

CI=[(SV×HR)/1000]/body⁢surface⁢area

Any additional CW Doppler waveforms not included in the consecutive SV analysis were analyzed for HR in the seated and standing posture where available.

#### 2.3.2. Blood pressure and systemic vascular resistance index

Systolic blood pressure was recorded using an Omron BP791IT 10 series Plus Automatic Blood Pressure Monitor with ComFitTM Cuff (Lake Forest, IL, USA) in a seated position (4-month and 8-month studies) and a standing position (8-month study only) on the non-dominant arm (Yamazaki et al., 2022b). Participants were seated (or standing) for 3 min before BP collection. The average value of three consecutive readings, taken 1 min apart, was used for analyses. SVRI was calculated by assuming that central venous pressure was zero and by using the following equation, whereby mean arterial pressure = (SBP + 2 × diastolic BP)/3 (Klabunde, 2012; Norsk et al., 2015; Yamazaki et al., 2022b):


SVRI=mean⁢arterial⁢pressure/CI

### 2.4. Statistical analyses

Data from the 4-month study (*N* = 6) and the 8-month study (*N* = 5) were pooled together for analysis across months 2 and 4 (*N* = 11). The additional 8-month time point data were collected in a subset of the N = 11 participants (*N* = 5). Intraclass correlation coefficients (ICCs) and their 95% confidence intervals (CIs) (two-way mixed, absolute agreement, average measures; SPSS v26, SPSS Inc., Chicago, IL, USA) assessed the interindividual differences and intraindividual stability of seated and standing CV measures (SBP, HR, SV, CI, LVET, and SVRI) at BL, TSD and REC across months 2, 4, and 8, and the average of BL and REC (BL + REC) across months 2, 4, and 8 (Tarokh et al., 2015). The following ranges characterize ICCs and reflect the stability of interindividual differences: 0.0–0.2 (slight); 0.2–0.4 (fair); 0.4–0.6 (moderate); 0.6–0.8 (substantial); and 0.8–1.0 (almost perfect) (Landis and Koch, 1977). As in prior studies (Spaeth et al., 2015; Dennis et al., 2017; Rusterholz et al., 2017; Yamazaki and Goel, 2020), Spearman’s rho (ρ) assessed the relative rank of individuals’ averaged BL-BL, TSD-TSD, and REC-REC responses across CV measures over 2 and 4 months (*N* = 11; SPSS v26, SPSS Inc., Chicago, IL, USA). Paired *t*-tests assessed differences in total sleep time (TST) for each BL and each REC night between months 2 and 4 in the combined sample (*N* = 11). Repeated measures (RM) ANOVAs compared TST for each BL and each REC night across months 2, 4, and 8 in the 8-month subset (*N* = 5). *Post hoc* analyses with Bonferroni corrections compared TST for a specific night across months 2, 4, and 8 when there was a significant time effect (e.g., TST during B1 at month 2 vs. TST during B1 at month 4 vs. TST during B1 at month 8). Any significant TST differences have been noted in Table 1. *P* < 0.05 was considered significant. SBP was collected in a standing position only in the 8-month study subset (*N* = 5). For the 8-month study subset, *N* = 1 participant was not included in the standing BL or BL + REC analyses due to incomplete standing data during month 8 at BL (*N* = 4). Additionally, because SBP was not taken in the standing position in the 4-month study, there are no data for standing SBP or SVRI in the combined 4-month study and 8-month study.

## 3. Results

### 3.1. Baseline (BL)

**FIGURE 1:**
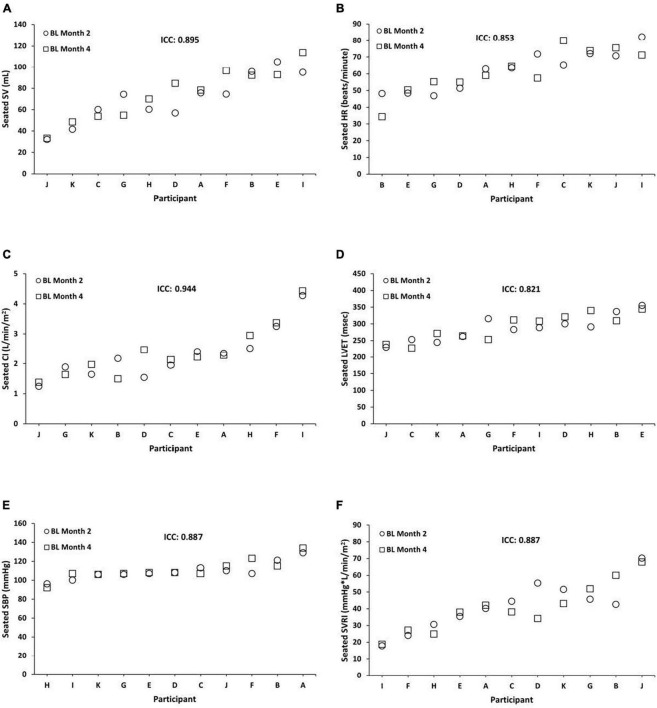
Individual differences and phenotypic stability of seated cardiovascular measures during baseline (BL). Seated BL ICCs were almost perfect across months 2 and 4 for the following (*N* = 11): **(A)** stroke volume (SV) = 0.895 (95% CI, 0.628, 0.971; *P* = 0.001); **(B)** heart rate (HR) = 0.853 (95% CI, 0.436, 0.961; *P* = 0.004); **(C)** cardiac index (CI) = 0.944 (95% CI, 0.801, 0.985; *P* = 0.000); **(D)** left ventricular ejection time (LVET) = 0.821 (95% CI, 0.307, 0.952; *P* = 0.008); **(E)** systolic blood pressure (SBP) = 0.887 (95% CI, 0.596, 0.969; *P* = 0.001); and **(F)** systemic vascular resistance index (SVRI) = 0.887 (95% CI, 0.576, 0.970; *P* = 0.001). Participants A, B, C, E, F, and G were male; participants D, H, I, J, and K were female. ICC, intraclass correlation coefficient; CI, confidence interval.

We examined the stability of seated CV measures at BL, TSD, and REC between months 2 and 4. Seated BL ICCs were almost perfect across months 2 and 4 for SV = 0.895 (95% CI, 0.628, 0.971; *P* = 0.001), HR = 0.853 (95% CI, 0.436, 0.961; *P* = 0.004), CI = 0.944 (95% CI, 0.801, 0.985; *P* = 0.000), LVET = 0.821 (95% CI, 0.307, 0.952; *P* = 0.008), SBP = 0.887 (95% CI, 0.596, 0.969; *P* = 0.001), and SVRI = 0.887 (95% CI, 0.576, 0.970; *P* = 0.001) (Figure 1). In exploratory analyses, we also examined ICCs for males and females separately for seated SV (male ICC = 0.830; female ICC = 0.920), HR (male ICC = 0.766; female ICC = 0.908), CI (male ICC = 0.920; female ICC = 0.959), LVET (male ICC = 0.838; female ICC = 0.858), SBP (male ICC = 0.826; female ICC = 0.904), and SVRI (male ICC = 0.805; female ICC = 0.929).

Similarly, standing BL ICCs were substantial to almost perfect across months 2 and 4 for SV = 0.909 (95% CI, 0.655, 0.976; *P* = 0.001), HR = 0.767 (95% CI, 0.125, 0.938; *P* = 0.018), CI = 0.920 (95% CI, 0.703, 0.978; *P* = 0.000), and LVET = 0.891 (95% CI, 0.593, 0.971; *P* = 0.001). In exploratory analyses, we also examined ICCs for males and females separately for standing SV (male ICC = 0.759; female ICC = 0.973), HR (male ICC = 0.861; female ICC = 0.410), CI (male ICC = 0.577; female ICC = 0.972), and LVET (male ICC = 0.887; female ICC = 0.876).

In the 8-month study subset (*N* = 5), seated CV measures at BL showed substantial to almost perfect stability [except for SBP] while standing CV measures at BL (*N* = 4) demonstrated fair to almost perfect stability across months 2, 4, and 8 (Table 2).

**TABLE 2:** Seated and standing cardiovascular measures for BL, TSD, and REC across months 2, 4, and 8 (*N* = 5).

CV measure	ICC	95% Confidence interval	*P*-value
**SV**			
Seated BL	0.932	0.698, 0.992	0.001
Standing BL	0.887*	0.299, 0.992	0.017
Seated TSD	0.863	0.417, 0.984	0.005
Standing TSD	0.962	0.825, 0.996	0.000
Seated REC	0.960	0.821, 0.996	0.000
Standing REC	0.920	0.616, 0.991	0.002
**HR**			
Seated BL	0.897	0.549, 0.988	0.003
Standing BL	0.579*	−1.258, 0.971	0.166
Seated TSD	0.944	0.707, 0.994	0.000
Standing TSD	0.851	0.308, 0.983	0.013
Seated REC	0.770	0.122, 0.972	0.014
Standing REC	0.932	0.700, 0.992	0.001
**CI**			
Seated BL	0.892	0.475, 0.988	0.006
Standing BL	0.906*	0.378, 0.994	0.013
Seated TSD	0.643	−0.247, 0.956	0.068
Standing TSD	0.899	0.559, 0.988	0.002
Seated REC	0.914	0.603, 0.990	0.002
Standing REC	0.671	−0.154, 0.960	0.052
**LVET**			
Seated BL	0.693	−0.659, 0.967	0.086
Standing BL	0.570*	−2.368, 0.972	0.191
Seated TSD	0.809	0.230, 0.977	0.009
Standing TSD	0.741	−0.203, 0.971	0.052
Seated REC	0.918	0.637, 0.991	0.002
Standing REC	0.894	0.481, 0.988	0.006
**SBP**			
Seated BL	0.198	−1.151, 0.890	0.338
Standing BL	0.376*	−1.789, 0.929	0.261
Seated TSD	0.670	−1.367, 0.965	0.112
Standing TSD	0.644	−1.138, 0.962	0.119
Seated REC	0.792	−0.207, 0.978	0.041
Standing REC	0.835	0.011, 0.982	0.025
**SVRI**			
Seated BL	0.939	0.715, 0.993	0.001
Standing BL	0.916*	0.451, 0.994	0.010
Seated TSD	0.649	−0.306, 0.958	0.075
Standing TSD	0.838	0.275, 0.982	0.014
Seated REC	0.922	0.639, 0.991	0.002
Standing REC	0.810	0.223, 0.978	0.015

*N = 4; BL, baseline; TSD, total sleep deprivation; REC, recovery; ICC, intraclass correlation coefficient; SV, stroke volume; HR, heart rate; CI, cardiac index; LVET, left ventricular ejection time; SBP, systolic blood pressure; SVRI, systemic vascular resistance index.

### 3.2. Total Sleep Deprivation (TSD)

**FIGURE 2:**
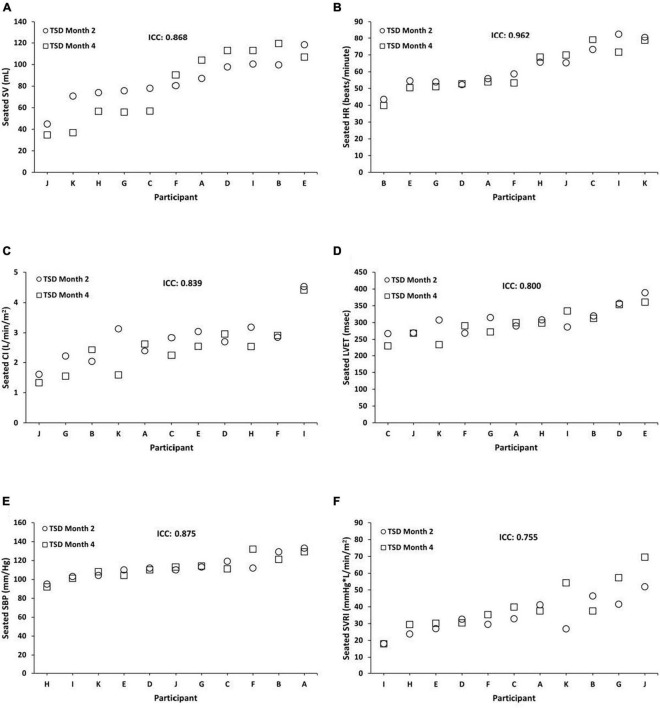
Individual differences and phenotypic stability of seated cardiovascular measures during total sleep deprivation (TSD). Seated TSD ICCs were substantial to almost perfect across months 2 and 4 for the following (*N* = 11): **(A)** stroke volume (SV) = 0.868 (95% CI, 0.513, 0.964; *P* = 0.002); **(B)** heart rate (HR) = 0.962 (95% CI, 0.867, 0.990; *P* = 0.000); **(C)** cardiac index (CI) = 0.839 (95% CI, 0.420, 0.956; *P* = 0.002); **(D)** left ventricular ejection time (LVET) = 0.800 (95% CI, 0.300, 0.945; *P* = 0.008); **(E)** systolic blood pressure (SBP) = 0.875 (95% CI, 0.520, 0.967; *P* = 0.002); and **(F)** systemic vascular resistance index (SVRI) = 0.755 (95% CI, 0.166, 0.933; *P* = 0.010). Participants A, B, C, E, F, and G were male; participants D, H, I, J, and K were female. ICC, intraclass correlation coefficient; CI, confidence interval.

Seated TSD ICCs were substantial to almost perfect across months 2 and 4 for SV = 0.868 (95% CI, 0.513, 0.964; *P* = 0.002), HR = 0.962 (95% CI, 0.867, 0.990; *P* = 0.000), CI = 0.839 (95% CI, 0.420, 0.956; *P* = 0.002), LVET = 0.800 (95% CI, 0.300, 0.945; *P* = 0.008), SBP = 0.875 (95% CI, 0.520, 0.967; *P* = 0.002), and SVRI = 0.755 (95% CI, 0.166, 0.933; *P* = 0.010) (Figure 2). In exploratory analyses, we also examined ICCs for males and females separately for seated SV (male ICC = 0.816; female ICC = 0.894), HR (male ICC = 0.966; female ICC = 0.931), CI (male ICC = 0.618; female ICC = 0.879), LVET (male ICC = 0.895; female ICC = 0.667), SBP (male ICC = 0.660; female ICC = 0.962), and SVRI (male ICC = 0.663; female ICC = 0.805).

Likewise, standing TSD ICCs were almost perfect across months 2 and 4 for SV = 0.958 (95% CI, 0.842, 0.989; *P* = 0.000), HR = 0.878 (95% CI, 0.571, 0.967; *P* = 0.001), CI = 0.954 (95% CI, 0.835, 0.987; *P* = 0.000), and LVET = 0.852 (95% CI, 0.453, 0.960; *P* = 0.003). In exploratory analyses, we also examined ICCs for males and females separately for standing SV (male ICC = 0.911; female ICC = 0.993), HR (male ICC = 0.947; female ICC = 0.797), CI (male ICC = 0.882; female ICC = 0.973), and LVET (male ICC = 0.837; female ICC = 0.879).

In the 8-month study subset (*N* = 5), seated and standing CV measures at TSD showed substantial to almost perfect stability across months 2, 4, and 8 (Table 2).

### 3.3. Recovery (REC)

**FIGURE 3:**
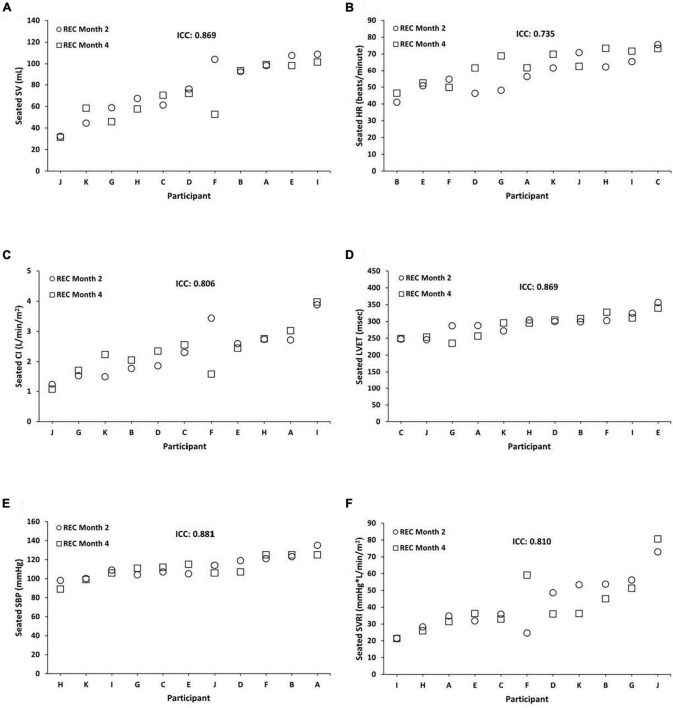
Individual differences and phenotypic stability of seated cardiovascular measures during recovery (REC). Seated REC ICCs were substantial to almost perfect across months 2 and 4 for the following (*N* = 11): **(A)** stroke volume (SV) = 0.869 (95% CI, 0.544, 0.964; *P* = 0.001); **(B)** heart rate (HR) = 0.735 (95% CI, 0.101, 0.927; *P* = 0.012); **(C)** cardiac index (CI) = 0.806 (95% CI, 0.241, 0.949; *P* = 0.010); **(D)** left ventricular ejection time (LVET) = 0.869 (95% CI, 0.518, 0.965; *P* = 0.002); **(E)** systolic blood pressure (SBP) = 0.881 (95% CI, 0.562, 0.968; *P* = 0.001); and **(F)** systemic vascular resistance index (SVRI) = 0.810 (95% CI, 0.257, 0.950; *P* = 0.010). Participants A, B, C, E, F, and G were male; participants D, H, I, J, and K were female. ICC, intraclass correlation coefficient; CI, confidence interval.

Seated REC ICCs were substantial to almost perfect across months 2 and 4 for SV = 0.869 (95% CI, 0.544, 0.964; *P* = 0.001), HR = 0.735 (95% CI, 0.101, 0.927; *P* = 0.012), CI = 0.806 (95% CI, 0.241, 0.949; *P* = 0.010), LVET = 0.869 (95% CI, 0.518, 0.965; *P* = 0.002), SBP = 0.881 (95% CI, 0.562, 0.968; *P* = 0.001), and SVRI = 0.810 (95% CI, 0.257, 0.950; *P* = 0.010) (Figure 3). In exploratory analyses, we also examined ICCs for males and females separately for seated SV (male ICC = 0.694; female ICC = 0.976), HR (male ICC = 0.799; female ICC = 0.379), CI (male ICC = 0.168; female ICC = 0.963), LVET (male ICC = 0.862; female ICC = 0.929), SBP (male ICC = 0.864; female ICC = 0.799), and SVRI (male ICC = 0.202; female ICC = 0.944).

Comparably, standing REC ICCs were almost perfect (except for LVET) across months 2 and 4 for SV = 0.952 (95% CI, 0.819, 0.987; *P* = 0.000), HR = 0.859 (95% CI, 0.507, 0.961; *P* = 0.002), CI = 0.918 (95% CI, 0.711, 0.978; *P* = 0.000), and LVET = 0.392 (95% CI, −1.679, 0.843; *P* = 0.239). In exploratory analyses, we also examined ICCs for males and females separately for standing SV (male ICC = 0.938; female ICC = 0.962), HR (male ICC = 0.915; female ICC = 0.739), CI (male ICC = 0.867; female ICC = 0.943), and LVET (male ICC = 0.331; female ICC = 0.502).

In the 8-month study subset (*N* = 5), seated and standing CV measures at REC showed substantial to almost perfect stability across months 2, 4, and 8 (Table 2).

### 3.4. Baseline + Recovery (BL + REC)

Seated BL + REC ICCs were almost perfect across months 2 and 4 for SV = 0.961 (95% CI, 0.856, 0.990; *P* = 0.000), HR = 0.883 (95% CI, 0.592, 0.968; *P* = 0.001), CI = 0.935 (95% CI, 0.759, 0.982; *P* = 0.000), LVET = 0.874 (95% CI, 0.517, 0.966; *P* = 0.002), SBP = 0.943 (95% CI, 0.786, 0.985; *P* = 0.000), and SVRI = 0.898 (95% CI, 0.614, 0.973; *P* = 0.001). In exploratory analyses, we also examined ICCs for males and females separately for seated SV (male ICC = 0.918; female ICC = 0.981), HR (male ICC = 0.856; female ICC = 0.861), CI (male ICC = 0.822; female ICC = 0.966), LVET (male ICC = 0.867; female ICC = 0.931), SBP (male ICC = 0.923; female ICC = 0.917), and SVRI (male ICC = 0.720; female ICC = 0.944).

Similarly, standing BL + REC ICCs were substantial to almost perfect across months 2 and 4 for SV = 0.965 (95% CI, 0.869, 0.991; *P* = 0.000), HR = 0.845 (95% CI, 0.400, 0.959; *P* = 0.005), CI = 0.955 (95% CI, 0.834, 0.988; *P* = 0.000), and LVET = 0.775 (95% CI, 0.111, 0.941; *P* = 0.017). In exploratory analyses, we also examined ICCs for males and females separately for standing SV (male ICC = 0.919; female ICC = 0.982), HR (male ICC = 0.913; female ICC = 0.650), CI (male ICC = 0.829; female ICC = 0.981), and LVET (male ICC = 0.779; female ICC = 0.782).

In the 8-month study subset (*N* = 5), seated BL + REC ICCs were moderate to almost perfect across months 2, 4, and 8 for SV = 0.952 (95% CI, 0.784, 0.995; *P* = 0.000), HR = 0.870 (95% CI, 0.435, 0.985; *P* = 0.003), CI = 0.948 (95% CI, 0.735, 0.994; *P* = 0.001), LVET = 0.552 (95% CI, −0.282, 0.940; *P* = 0.085), SBP = 0.712 (95% CI, −0.326, 0.968; *P* = 0.065), and SVRI = 0.965 (95% CI, 0.837, 0.996; *P* = 0.000). Standing BL + REC ICCs in the 8-month study subset (*N* = 4) were substantial to almost perfect across months 2, 4, and 8 for SV = 0.952 (95% CI, 0.740, 0.997; *P* = 0.001), HR = 0.899 (95% CI, 0.440, 0.993; *P* = 0.010), CI = 0.894 (95% CI, 0.448, 0.993; *P* = 0.009), LVET = 0.819 (95% CI, −0.203, 0.988; *P* = 0.047), SBP = 0.759 (95% CI, −0.179, 0.973; *P* = 0.047), and SVRI = 0.965 (95% CI, 0.784, 0.998; *P* = 0.000).

### 3.5. Cardiovascular measures: relative rank relationships

Individuals also exhibited significant consistency of responses within seated and standing CV measures across months 2 and 4 during BL, TSD, and REC. For seated measures during BL (Table 3), SV was positively correlated with CI (ρ = 0.682, *P* = 0.021) and LVET (ρ = 0.755, *P* = 0.007). HR was negatively correlated with LVET (ρ = −0.645, *P* = 0.032) and CI was negatively correlated with SVRI (ρ = −0.964, *P* = 0.000). There were no other significant correlations. For standing measures during BL, SV was positively correlated with CI (ρ = 0.764, *P* = 0.006). There were no other significant correlations.

**TABLE 3:** Spearman’s rank correlation coefficients for seated cardiovascular measures for baseline (BL).

	SV	HR	CI	LVET	SBP	SVRI
**SV**		-0.336	0.682*	0.755**	0.064	-0.600
**HR**	-0.336		0.164	-0.645*	-0.282	-0.227
**CI**	0.682*	0.164		0.391	-0.136	-0.964**
**LVET**	0.755**	-0.645*	0.391		-0.191	-0.345
**SBP**	0.064	-0.282	-0.136	-0.191		0.309
**SVRI**	-0.600	-0.227	-0.964**	-0.345	0.309	

*N* = 11; **P < 0.01, *P < 0.05; SV, stroke volume; HR, heart rate; CI, cardiac index; LVET, left ventricular ejection time; SBP, systolic blood pressure; SVRI, systemic vascular resistance index.

For seated measures during TSD (Table 4), SV was positively correlated with LVET (ρ = 0.791, *P* = 0.004). HR was negatively correlated with LVET (ρ = −0.627, *P* = 0.039) and CI was negatively correlated with SVRI (ρ = −0.936, *P* = 0.000). There were no other significant correlations. For standing measures during TSD, SV was negatively correlated with HR (ρ = −0.664, *P* = 0.026) and positively correlated with LVET (ρ = 0.827, *P* = 0.002), and HR was negatively correlated with LVET (ρ = −0.673, *P* = 0.023). There were no other significant correlations.

**TABLE 4:** Spearman’s rank correlation coefficients for seated cardiovascular measures for total sleep deprivation (TSD).

	SV	HR	CI	LVET	SBP	SVRI
**SV**		-0.573	0.391	0.791**	0.182	-0.455
**HR**	-0.573		0.209	-0.627*	-0.436	-0.145
**CI**	0.391	0.209		0.355	-0.382	-0.936**
**LVET**	0.791**	-0.627*	0.355		-0.209	-0.509
**SBP**	0.182	-0.436	-0.382	-0.209		0.545
**SVRI**	-0.455	-0.145	-0.936**	-0.509	0.545	

*N* = 11; **P < 0.01, *P < 0.05; SV, stroke volume; HR, heart rate; CI, cardiac index; LVET, left ventricular ejection time; SBP, systolic blood pressure; SVRI, systemic vascular resistance index.

For seated measures during REC (Table 5), SV was positively correlated with CI (ρ = 0.747, *P* = 0.008) and LVET (ρ = 0.738, *P* = 0.010), and negatively correlated with SVRI (ρ = −0.629, *P* = 0.038). CI was negatively correlated with SVRI (ρ = −0.973, *P* = 0.000). There were no other significant correlations. For standing measures during REC, SV was positively correlated with CI (ρ = 0.700, *P* = 0.016) and LVET (ρ = 0.682, *P* = 0.021) and CI was positively correlated with LVET (ρ = 0.736, *P* = 0.010). There were no other significant correlations.

**TABLE 5:** Spearman’s rank correlation coefficients for seated cardiovascular measures for recovery (REC).

	SV	HR	CI	LVET	SBP	SVRI
**SV**		-0.305	0.747**	0.738**	0.351	-0.629*
**HR**	-0.305		0.191	-0.509	-0.591	-0.345
**CI**	0.747**	0.191		0.500	-0.018	-0.973**
**LVET**	0.738**	-0.509	0.500		0.073	-0.418
**SBP**	0.351	-0.591	-0.018	0.073		0.218
**SVRI**	-0.629*	-0.345	-0.973**	-0.418	0.218	

*N* = 11; **P ≤ 0.01, *P < 0.05; SV, stroke volume; HR, heart rate; CI, cardiac index; LVET, left ventricular ejection time; SBP, systolic blood pressure; SVRI, systemic vascular resistance index.

## 4. Discussion

We found robust phenotypic stability of a number of CV measures in healthy adults during BL, TSD, and REC across 2-month, 4-month, and 8-month time points. Seated CV measures showed substantial to almost perfect ICCs at BL, TSD, and REC across months 2, 4, and 8. Seated CV measures also showed moderate to almost perfect stability for the average of BL and REC (BL + REC) across months 2, 4, and 8. In addition, individuals demonstrated significant consistency of responses within seated CV measures across months 2 and 4 during BL, TSD, and REC: SV was positively correlated with CI and LVET while negatively correlated with SVRI, HR was negatively correlated with LVET, and CI was negatively correlated with SVRI. Standing CV measures showed similar ICCs and consistency of responses. For the first time, we demonstrate long-term robust phenotypic stability of CV measures in healthy adults during repeated BL, TSD, and REC exposures. Cardiovascular measures may serve as biomarkers given they can track cardiovascular physiology consistently across repeated acute sleep loss and recovery and across long duration. Our results demonstrated a higher seated HR ICC during TSD across 2 and 4 months (0.962) as well as across 2, 4, and 8 months (0.944) compared to a laboratory study of *N* = 12 adults, which found long-term HR stability (ICC = 0.790) after exposure to 26 h of TSD across 2.5–15 months (Chua et al., 2014). The higher HR ICCs during TSD in our results may be due to our studies’ isolated environment, longer acute TSD duration, or method of HR collection via echocardiography as compared to the previous study, which collected HR via electrocardiogram (ECG). In addition, our studies are the first to examine the stability of SBP, SV, CI, LVET, and SVRI after exposure to total sleep deprivation, which demonstrated substantial to almost perfect stability (0.643–0.962).

The stability of various CV measures at BL during fully rested conditions across months 2, 4, and 8 is also comparable to research on both short-term and long-term stability across repeated collections of CV measures that did not involve sleep deprivation (Woodward et al., 1995; Mier et al., 1997; Rowland et al., 1998; Goedhart et al., 2006; Hinderliter et al., 2013; Legendre et al., 2018). Our results at BL almost without exception showed higher seated ICCs for SV (0.895), HR (0.853), CI (0.944), LVET (0.821), and SBP (0.887) across 2 and 4 months compared to studies of short-term and long-term stability of CV measures including SV (0.290–0.800), HR (0.500–0.920), CO (0.330–0.930), LVET (0.520–0.770), and SBP (0.030–0.860), which used a span of 2 h to 40 months (Woodward et al., 1995; Mier et al., 1997; Rowland et al., 1998; Goedhart et al., 2006; Hinderliter et al., 2013; Legendre et al., 2018). Our higher ICCs may be due to our studies’ highly controlled environment as compared to past studies, which occurred in mostly outpatient settings. Other factors may have affected the lower stability reported in other studies such as exercise or CV measure collection methods including ECG, impedance cardiography, thoracic bioelectrical impedance, and the Collier re-breathing method, compared to our studies’ CV measure collection method via echocardiography and BP monitor.

Our findings are also comparable to past research that has examined the stability of various other measures across repeated exposures to sleep loss (Tucker et al., 2007; Tarokh et al., 2015; Dennis et al., 2017; Rusterholz et al., 2017; Hennecke et al., 2019; Ong et al., 2019; Yamazaki and Goel, 2020). Stable and trait-like interindividual differences have been observed in polysomnographic sleep and slow-wave energy responses to TSD across 2–3 days, as well as electroencephalogram power spectra responses to SR and 1-h naps (Tucker et al., 2007; Tarokh et al., 2015; Rusterholz et al., 2017; Ong et al., 2019). Research has also found robust stability across exposures and long-time intervals to SR and TSD in various neurobehavioral performance measures (Dennis et al., 2017; Yamazaki and Goel, 2020). In addition, robust stability of polysomnography has been shown across 3 nights of BL, across 3 nights of REC, as well as across BL and REC nights combined (BL + REC) after 36 h of TSD (Tarokh et al., 2015), and between one night of BL and REC after 58 h of TSD (Hennecke et al., 2019).

Of interest, the ICCs of the standing CV measures were within the ranges of the seated CV measures indicating that all participants were at equivalent baseline volume status in the two postural conditions. Standing ICCs at BL, TSD, and REC showed substantial to almost perfect stability for most CV measures, and BL + REC ICCs showed substantial to almost perfect stability across months 2, 4, and 8. Individuals also exhibited similar consistency of responses in both seated and standing CV measures, with a few minor exceptions across months 2 and 4 during BL, TSD, and REC, which may be due to sleep loss differentially affecting some variables and not others across postural positions. Of note, when comparing BL vs. REC, ICCs (not reported in the main results) for seated and standing CV measures maintained moderate to almost perfect stability at month 2, at month 4, and at month 8 (except for LVET). These results were within the ranges of seated and standing BL ICCs, REC ICCs, and BL + REC ICCs across months 2, 4, and 8.

In exploratory analyses, we examined the stability of CV responses by sex at BL, TSD, and REC. For many of the CV measures across these phases, males and females showed similar stability; however, males were more stable on certain measures such as HR at BL, REC, and BL + REC and females were more stable on other measures including CI at BL, TSD, and REC, SVRI at TSD and REC, and SBP at TSD. These differences in CV stability may be due to fluctuations within the menstrual cycle (Girdler et al., 1990, 1993) or other factors including our small sample size. In our other studies involving sleep deprivation and stability, we also observed sex differences in daily caloric intake and weight change (Spaeth et al., 2015; Dennis et al., 2016), but not in neurobehavioral performance and late-night eating (Spaeth et al., 2015; Yamazaki and Goel, 2020). Considering sex differences reported in many cardiovascular measures (Girdler et al., 1990, 1993), our results should be replicated in future larger studies examining sex differences in CV stability.

Individuals who are sleep deprived due to work obligations such as staying up late for shift work or lifestyle choices and who exhibit phenotypic vulnerability to the CV effects of sleep loss are at particularly heightened risk for developing CV and associated diseases. Examining the stability of CV indices after exposure to sleep deprivation is vital to the recommendation of mitigation strategies related to real-world settings such as transportation (Mahajan and Velaga, 2021) and emergency services (Jahnke et al., 2012), and the military (Seelig et al., 2016; Shattuck et al., 2018), among others, thus further adding to the criticality of such research.

While definitive CV biomarkers for the prediction of vulnerability in sleep deprivation have yet to be discovered, genetic and omics (e.g., transcriptomic, epigenomic, proteomic, and metabolomic) approaches have identified biomarkers to distinguish an individual’s response to TSD (Goel, 2015, 2017; Casale and Goel, 2021). This is the first evidence of phenotypic trait-like stability of CV responses to repeated exposure to sleep deprivation: our findings open the door for biomarker discovery and countermeasure development to mitigate and predict this vital health-related vulnerability in both fully rested and sleep deprivation conditions.

There are a few limitations to our studies. One limitation was the small sample size. In addition, because of the small sample size, the examination of potential sex differences in stability of CV measures can only be considered exploratory. Furthermore, it is difficult to generalize our findings to individuals with mood or sleep disorders, or with other medical conditions, since all participants in our studies were healthy adults who were not shift workers or exposed to TSD in their professions. We also note that physical and cognitive activity level, meal quality, quantity, and timing, as well as water intake were not strictly controlled. Our results may also not be generalizable to circumstances that do not involve isolation; notably, however, isolation was required for our experiments investigating hemodynamic changes in high-fidelity space analogs to simulate spaceflight conditions (Le Roy et al., 2023). Moreover, in our studies, VTI of the CW wave form of the ascending aorta was used as a surrogate measure for LVOT VTI.

For the first time, we demonstrate robust phenotypic stability of CV measures in healthy adults during BL, TSD, and REC across 2-month, 4-month, and 8-month time points in long-duration studies. We also demonstrate stability of other CV biomarkers in healthy adults never examined previously during sleep deprivation and recovery including SBP, SV, CI, LVET, and SVRI. In addition, individuals showed significant consistency of responses within CV measures in long-duration studies. Overall, our results herald the use of the CV biomarkers examined in our studies, including SV, HR, CI, LVET, SVRI, and SBP, and countermeasures for prediction and mitigation of this critical vulnerability in both fully rested and sleep deprivation conditions.

## Data availability statement

The data generated and analyzed during the current study are available from the corresponding author upon reasonable request.

## Ethics statement

The studies involving human participants were reviewed and approved by the Institutional Review Board of the NASA (4-month study and 8-month study) with primary oversight and also by the Institutional Review Boards of the University of Pennsylvania (4-month study) and Rush University Medical Center (8-month study). The participants provided their written informed consent to participate in this study.

## Author contributions

NG designed the overall study and provided the financial support. LP conducted the statistical analyses of the data. KR-G extracted and analyzed the echocardiography data. All authors prepared the manuscript and reviewed and approved the final manuscript.
